# When and Why People Prefer Higher Educated Politicians: Ingroup Bias, Deference, and Resistance

**DOI:** 10.1177/01461672221077794

**Published:** 2022-02-22

**Authors:** Jochem van Noord, Toon Kuppens, Bram Spruyt, Russell Spears

**Affiliations:** 1 University of Groningen, The Netherlands; 2 Vrije Universiteit Brussel, Belgium

**Keywords:** education, social identity, competence, politics, ingroup bias

## Abstract

When choosing between political candidates of different educational levels, do voters show ingroup bias or base their vote choice on candidates’ perceived competence? We aim to investigate how (fictional) political candidates of different educational levels are evaluated and voted for, how this is affected by voters’ educational level, and the role of perceived (Study 1) and manipulated competence (Study 2). Higher educated participants preferred higher to less educated candidates over and above their level of competence, particularly when they identified strongly with their educational level. This reflects ingroup bias among the higher educated. Less educated participants preferred higher educated candidates in Study 1, but did not prefer higher educated candidates when competence was manipulated independently from education in Study 2. The less educated, unlike the higher educated, therefore, seem to show deference to the assumed competence of the higher educated, because it disappears when more reliable competence information is available.

A recurring question in intergroup relations is whether low-status groups challenge the status quo, accept their inferior position and defer to the high-status group, or even actively support the hierarchy. A topical example of this is the place of the less educated in the political arena. When voting for political candidates, do less educated voters show ingroup bias and prefer “one of their own” over higher educated candidates?^1^ Or do they defer to the supposed superior (cognitive or academic) competence of the higher educated (Baker, 2014; Spruyt & Kuppens, 2014) and prefer a higher educated candidate? For higher educated voters, there is no such dilemma because both possible ingroup bias and the desire to pick a competent candidate point in the same direction and should lead them to prefer higher educated politicians. However, for the higher educated, the question then becomes whether candidate competence or ingroup bias matters more for vote choice. The rise of populism investigating the processes underlying the choice of political leaders is topical and timely.

One reason to expect education-based ingroup bias in voting preferences is that education (and not income) plays an important role in current political conflicts. Populist radical-right parties criticize technocratic elements of modern politics and often appeal to the (less educated) “common man” (sic; Mudde, 2004; Rooduijn, 2014). In general, the less educated are in many countries^2^ indeed more likely to support these parties and their anti-immigration policies, and this is, demographically speaking, related to their educational level, rather than income or occupation (Rooduijn, 2017; Spruyt et al., 2016). In contrast, others have argued for a *more* technocratic nature of democracy. For example, some have proposed that the right to vote should depend on political knowledge, effectively excluding many, and mostly, less educated citizens (Bell, 2016; Brennan, 2016). In other words, education (and not income) is central to a major current political divide, both in terms of voter behavior and political rhetoric (Evans & Tilley, 2017; Zhang, 2018).

However, one area where this educational conflict is paradoxically absent is in the educational level of political representatives. Indeed, one reason why our central question is particularly pertinent is because political representation in Western societies is increasingly dominated by the higher educated. For example, in six West European countries, the higher educated occupy between 75% and 95% of the parliamentary seats, the result of a steady increase over the past 90 years (Bovens & Wille, 2017, p. 114). Furthermore, few (if any) of the elected radical-right leaders are themselves less educated. This constitutes an education paradox in current politics: There is an education-based conflict in voter behavior and political rhetoric, but the less educated are increasingly underrepresented in political positions of power. The reasons for this paradox remain largely unexplored.

Over two studies with nationally representative samples from the Netherlands, we presented participants with fictitious political candidates whose educational level, political preferences, and competence were manipulated. We aim to answer two crucial questions. What educational background do higher and less educated voters prefer for candidate selection? And how can we best explain these preferences?

## Education-Based Status

Earlier research into the effect of politicians’ educational background on voting preferences has produced conflicting evidence. Several studies found a general preference for higher educated candidates (Arnesen et al., 2019; Hainmueller et al., 2014; Wüest & Pontusson, 2017). However, one study found no distinction between higher and less educated candidates (Carnes & Lupu, 2016), and another observed a voting preference for less educated candidates (Campbell & Cowley, 2014). That research has found no clear preference for higher educated candidates is notable, because in the real world the higher educated clearly dominate political representation. Furthermore, the higher status of the higher educated in western societies is based on their assumed competence and intelligence (Baker, 2014; Van Noord et al., 2019), and this “academic competence ideology” can be a reason to prefer higher educated candidates. Theories of educational systems in modern western societies posit that the educational system has taken a central authoritative role in shaping culture and institutions in such a way that it defines society’s winners and losers (Baker, 2014; Meyer, 1977). This categorization is based on the success people attain in the educational system, which focuses heavily on (cognitive) competence. Cognitive competence or intelligence is assessed in individual tests, which create the impression of real and legitimate individual differences (Goudeau & Croizet, 2017; Townsend et al., 2019).

As such, education is an *independent* axis of social stratification and cultural distinction, which cannot be reduced to income or (economic) class. Indeed, political research often finds effects of education that are different from the effects of income (e.g., Cavaille & Marshall, 2019; Rooduijn, 2017; Spruyt et al., 2016).

## Education-Based Conflict

The question remains whether this academic competence ideology is equally supported among all (educational) groups in society. Educational groups may take opposing positions, with voting driven by group interests. Particularly, the less educated are less likely to accept, and defer to, the supposed superior competence of the higher educated, as that would involve also accepting their own supposed *inferior* competence. Much evidence in intergroup relations testifies that people are likely to favor their ingroup (Tajfel & Turner, 1979). Indeed, in one study, the less educated, on average, did not see the higher educated as more competent, and when they identified strongly as less educated they saw the less educated as *more* competent than the higher educated (Spruyt & Kuppens, 2014). On this basis, the less educated should then prefer less educated politicians to represent them and their shared interests (Tajfel & Turner, 1979).

Indeed, recent research has pointed toward the growing awareness of educational identities in societies where the educational system takes a more central role (Gidron & Hall, 2019; Spruyt & Kuppens, 2015; Stubager, 2009; Van Noord et al., 2019). In general, people find their education as important to their sense of who they are as their age and more important than their ethnic background (Easterbrook et al., 2019). Furthermore, education can be grounds for stereotyping and intergroup bias, and these processes are generally stronger for those who identify stronger with their educational level (Kuppens et al., 2018). This renders it plausible that higher and less educated voters will be motivated by group concerns relating to these social identities.

However, there are also two reasons why ingroup bias might be *lower* among the less educated in particular. First, there is generally a relatively lower level of group identification among the less educated than among the higher educated (Stubager, 2009). The label of less educated carries a stigma: It mainly denotes having been unsuccessful in education, leading to lower identification, especially on group-esteem-related aspects of identity (Kuppens et al., 2015, p. 1261). It is possible that some less educated individuals prefer to identify with a different label such as working class (Tajfel & Turner, 1979), reflecting disidentification with their education-based identity. So while identification for higher educated is relatively strong, it remains uncertain whether this lower group identification provides a reliable basis for group-based identity and action for the less educated.

Second, most people perceive the educational system and its outcomes as *legitimate* (Kluegel & Smith, 1986; Van Noord et al., 2019). When differences are legitimate, low-status groups are less likely to show ingroup bias (Tajfel & Turner, 1979). This is also in line with system justification theory (Jost & Banaji, 1994) and the social identity model of system attitudes (Owuamalam et al., 2018), which both point to the possibility that low status groups may display behavior which effectively legitimizes and reproduces the status quo, even to the detriment of their own apparent interests. In sum, while there are compelling reasons to expect ingroup bias in education-based groups, there are also good arguments for why the less educated may *not* show such ingroup bias and opt to defer to the status of higher educated individuals. This article investigates which of these two contrasting predictions finds most empirical support.

For the higher educated, as the more dominant group, such cross-pressures are largely irrelevant. Because they are considerably more likely than the less educated to identify with their group, group-based preferences and action are more likely to arise for them. In addition, their status in society benefits from an academic competence ideology positioning them as more competent—encouraging their belief in these ideologies (Abercrombie et al., 1980; Jackman, 1994; Sidanius & Pratto, 2001). Expectations based on ingroup bias or on academic competence ideology thus point in the same direction: The higher educated are likely to favor higher educated political candidates who they are likely to see as more competent.

Furthermore, we expect that the relationships that revolve around ingroup bias are moderated by identification with people’s educational level (Spruyt & Kuppens, 2014, 2015) Although belief in academic competence ideologies is not likely to be dependent on how strongly one identifies with their educational level, showing ingroup bias is as previous research has found such moderations for educational groups (Kuppens et al., 2018; Spruyt & Kuppens, 2014). Thus, for both higher and less educated, we expect that preferences for one’s own group over the other group are moderated by identification. Higher educated who identify strongly would, thus, have a stronger preference for higher educated than those who identify weakly. Less educated who identify strongly would have a stronger preference for less educated or, a weaker preference for higher educated, than those who identify weakly.

## Research Overview

We investigate whether the preferences of voters from different educational groups for political candidates of different educational groups differ, and if so why? In two studies,^3^ we presented participants with four different profiles of political candidates that differed in their educational level (both studies), political orientation (Study 1), and competence (Study 2). Participants rated these candidates on competence, but also warmth (Fiske et al., 2002), agency (Koch et al., 2016), and morality (Leach et al., 2007) (in Study 1) to investigate whether effects are restricted to competence or generalize to other positive traits,^4^ or different aspects of competence (Study 2) to test whether education is still used by all as a marker of competence *even when* actual competence is additionally manipulated. We also asked participants to indicate the extent to which they feel a shared identity with their candidate (Study 1 only) and their voting preferences for each candidate (both studies). It is important to note here that this experiment is not necessarily a realistic simulation of political elections—the main aim is to understand processes around education-based status and identity in a political context, rather than simulating election processes. In addition, we measured identification with one’s educational group to investigate whether we find more evidence for intergroup processes such as ingroup bias among participants who are more highly committed to their education group.

We report all measures, manipulations, and exclusions in these studies. All measurements included in the studies but not reported in this text are reported on in the Supplemental Material. We also report all cell means, *SD*s, *n*s, and correlations between the measurements in the Supplemental Material. Sample sizes were determined before any data analysis.

## Study 1

In the first study, we used a nationally representative sample of the Netherlands to investigate whether higher educated candidates are evaluated as more competent than less educated candidates and whether this difference in evaluation is stronger among participants with a higher educational level or with a lower educational level. To reiterate, less educated participants could either have a preference for less educated candidates, resulting from an education-based group conflict (and associated ingroup bias), or have a preference for higher educated, due to their perceived higher competence, as informed by the consensually perceived legitimate status of higher educated (academic competence ideology).

### Method

Participants rated four fictitious political candidates who varied in educational level and political preference. This study, therefore, had a 3 (participant education: high, middle, and low), by 2 (candidate education: high vs. low), by 2 (candidate political preference: progressive vs. conservative) design, with the last two factors varying within participants.^5^ The study revolves partly around processes of identification. Identification might be more difficult if gender differs between participant and candidate. Letting gender vary within participants would complicate the design and require a larger sample size. To circumvent both problems, we matched the gender of the political candidate to participant gender.

#### Participants and power calculation

There are several focal analyses in Study 1, and we use multilevel analyses that are not easily used in power calculations, but we nevertheless did an approximate power calculation. With a conservative estimate of the effect size based on the pilot study (see Supplemental Material, Appendix 1), the required sample size for an attenuated interaction in a repeated-measures model is 702 (see Supplemental Appendix 2 for more details).

About 1,121 participants filled in the complete survey. Of these, 426 were removed due to not passing the attention check question (“Choose agree strongly if you have read this question”).^6^ The final sample consists of 695 participants (466 women, mean age = 44.0, *SD* = 12.1). Sensitivity analyses with the same assumptions as the power analysis above (except correlations among repeated measurements, which in this study is .5) give a minimum effect size of *f* = .050.

#### Education

Education was measured with eight categories that were collapsed into three: less educated (International Standard Classification of Education [ISCED] levels 0–2; 21.5%), middle educated (ISCED levels 3–5; 40.2%), and higher educated (ISCED levels 6–8; 38.3%). In the analyses, this variable is used as a categorical/factor variable. Both the labels (less/middle/higher) and the corresponding ISCED categorization are the conventional ways of categorizing educational levels in the Netherlands (i.e., it is the educational categorization used by the Dutch National Bureau of Statistics, CBS). Although we have clear expectations for the less and higher educated, this is not the case for the middle educated—therefore when describing the effects of participant education, we focus on the less and higher educated. Results for the middle educated are included in the regression models (see Supplemental Material).

#### Candidate education

The presented candidate profiles had either higher (master’s degree) or lower (high school diploma) educational qualifications. To increase the visibility of this manipulation, we described activities that the candidates did during their time at either the university or high school. We used two versions of the manipulations of education and political orientation, so that manipulated text was not repeated over the different profiles. Example manipulation text (less educated): “[candidate name] has a high school diploma, and was, during his/her time in high school active in the korfball association.” See Supplemental Appendix 3 for full education manipulation text.

#### Candidate political orientation

Because educational differences align with important cleavages in politics that are also marked by substantive differences, we also varied the candidates on political orientation. They were either presented as progressive (indicated by a priority for climate change and sustainability) or as conservative (indicated by a priority for law and order). Example manipulation text (conservative): “[Candidate name] thinks safety is an important issue. [Candidate name] thinks that criminals should be punished much harder and without reservations.” Previous research indeed shows that the largest educational differences are associated with this, sometimes called “cultural,” political dimension. Educational differences are usually much smaller when it concerns the economic political dimension which is dominated primarily by income differences (Achterberg & Houtman, 2006; Van der Waal et al., 2007). This manipulation controls for (assumed) substantive political positions. Results of this manipulation were not central to our article, and we do not report them here. See Supplemental Appendix 3 for full political orientation manipulation text and a report of the findings for this manipulation.

#### Candidate profile filler information

To improve realism, the profiles also contained random, but equivalent, filler information on name, date of birth, place of residence, marital status and children, and hobbies. See Supplemental Appendix 4 for the profile (filler) text.

#### Candidate perceptions

We asked participants to rate four political candidates on a total of eight characteristics (two per stereotype dimension) associated with competence (correlation between the two items: *r* = .77), warmth (*r* = .81), agency (*r* = .76), and morality (*r* = .80) and their willingness to vote for each of these candidates. In addition, we measured with two items (*r* = .89, example item: “to what extent do you think you can identify with this candidate?”) to what extent the participants identified with the presented candidates (feeling of shared identity). See Supplemental Appendix 5 for all stereotypes and shared identity items.

#### Educational identification

We measured identification with 10 items (α = .91, items are listed in Supplemental Appendix 6) adapted from Leach et al. (2008). This scale is validated in Leach et al. (2008) and has been used for a wide range of identities since then (including education-based identity, for example, Kuppens et al., 2015, 2018)

#### Control variables

We include age, gender, and three variables on political preferences: environmentalism (four items, α = .83), law and order (four items, α = .65), and ethnic prejudice (four items, α = .93). The demographic variables correlate with political preferences, and together with the political preference variables explain, for our current goal, an irrelevant part of the variance. We listed all items in Supplemental Appendix 7.

#### Procedure

Participants were first presented with the age, gender, and education^7^ questions. They were then presented with the four profiles and indicated for each profile their assessment of these candidates on the stereotype traits. After reviewing all four profiles, they were asked to express their vote intention toward these candidates. Finally, we asked them to complete the measures of educational identification, political orientation, and other measures that are not relevant here (listed in the Supplemental Material).

### Analytical Strategy

As we present all participants with four profiles to be assessed, we conduct multilevel models with the candidates (profiles) as the first level and participants as the second level in all our analyses. We use the repeated measures option of Stata’s—mixed—command to model the residuals with an unstructured covariance matrix at the level of the participants.

To aid interpretation, we have mean-centered and divided by *two times* the standard deviation (Gelman, 2008) all continuous (independent and dependent) variables (except age, which is divided by 10 and mean-centered). In this way, the coefficients of the binary (e.g., manipulations) and continuous (e.g., stereotypes) independent variables used in this study are directly comparable due to similar standard deviations. Coefficients of both binary and continuous variables can be interpreted similarly with the coefficient corresponding to the *SD* change in the dependent variable when the independent variable changes 1 *SD*. Since binary variables normally have a range of 2 *SD*, their coefficients will be twice as small as with the traditional way of standardization. See Gelman (2008) for more details. All labels in figures and reported means are on the original scales.

All our models are built stepwise rather than fully factorial. Model 1, thus, contains only the interaction term between candidate education and candidate political orientation, and control variables (age, gender, environmentalism, law and order, and ethnic prejudice). Other models build on this model. In Supplemental Appendix 9, we list all models and their specifications. We tested separately whether the higher order interactions that were not part of our hypotheses (e.g., Participant Education × Educational Identification × Candidate Education × Candidate Political Orientation) were significant, all but three coefficients (out of 72) of these interactions were nonsignificant. None of the significant interactions were of theoretical interest, but we listed them in Supplemental Appendix 10. We have included the regression tables in the Supplemental Material.

### Results

As we built the models stepwise, we started with a model containing only the candidate education, candidate political orientation, and their interaction, with the different stereotypes and vote intention as the dependent variables. In each section, we built on this model and added the relevant interaction: first participant education as a two-way interaction, then later also educational identification and its interactions as a three-way interaction. All models include the control variables mentioned above.

#### Candidate and participant education

Higher educated candidates were seen as more competent (*b* = .282, *p* < .001)^8^ and participants indicated higher vote intentions toward them (*M*_less educated candidate_ = 3.88, *M*_higher educated candidate_ = 4.21; *b* = .098, *p* < .001). This was moderated by participant education for both competence (*b* = .175, *p* < .001; simple effects: *b*_LE_ = .225, *p* < .001; *b*_HE_ = .400, *p* < .001), and vote intention (*b* = .133, *p* < .001; simple effects: *b*_LE_ = .070, *p* = .019; *b*_HE_ = .203; *p* < .001), with higher educated participants showing stronger effects of candidate education. Table 1 shows the (predicted) means for these effects. These indicate that the interaction effect for competence is mostly due to the higher educated participants rating less educated candidates as less competent than the less educated participants do. However, for vote intention, the interaction effect is driven by higher educated participants both being more negative about the less educated candidates, as more positive about the higher educated candidates. Furthermore, participants rated higher educated candidates higher on agency and morality, but not on warmth. No significant moderations by participant education were found for these stereotypes (see Supplemental Appendix 5 for results of stereotypes). Hence, participant education seems to only be relevant for competence ratings and vote intention.

**Table 1.** Coefficients of Candidate Education and Candidate Competence on Dimensions of Perceived Competence.

Competence	Candidate education			Candidate competence		
	*b*	*SE*	*p*	*b*	*SE*	*p*
Composite scale	.292	.013	.000	.238	.010	.000
Practical	.168	.014	.000	.308	.013	.000
Theoretical	.438	.015	.000	.193	.010	.000
Rhetorical	.359	.014	.000	.178	.010	.000
Social	.058	.013	.000	.102	.012	.000
Strategic	.208	.014	.000	.243	.013	.000

*Note.* Coefficients denote standardized (with *M* = 0, *SD* = 0.5) coefficients, and a *b*-coefficient of .5 thus means that there is a difference of 1 *SD* of the dependent variable between the two conditions. Candidate education/competence is dichotomous, where 1 means more educated/competent. *SE* = standard error.

**Figure 1.**
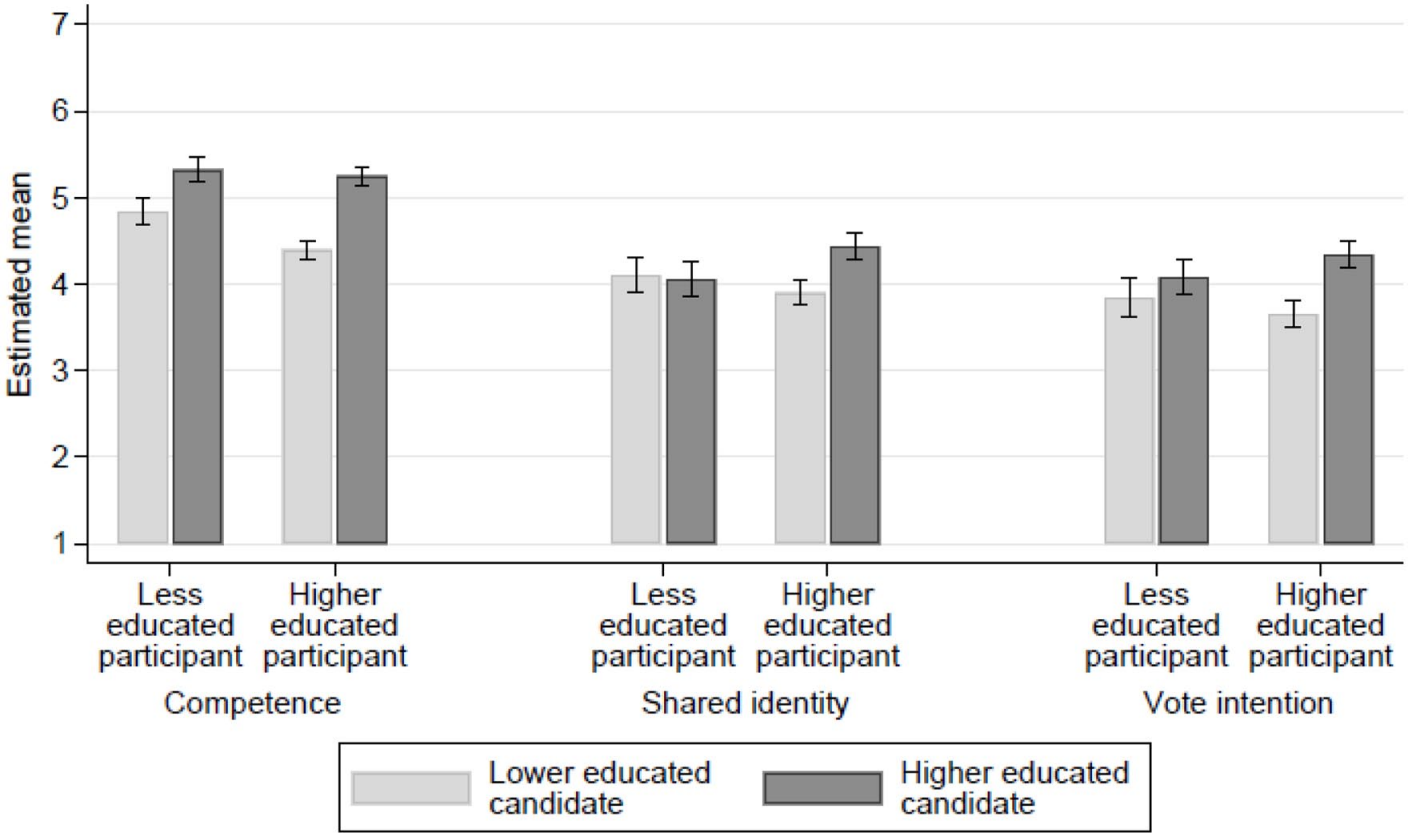
Predicted means of competence, shared identity, and vote intention across participant and candidate education. *Note.* Predicted means taken from model with Participant × Candidate Education, including control variables. Error bars denote 95% confidence intervals. Y-axis values are unstandardized values.

For feelings of shared identity with the candidate, there was an interaction between candidate education and participant education (*b* = .197, *p* < .001). Higher educated participants identified more strongly with higher educated candidates than with lower educated candidates (*b* = .181, *p* < .001; see Figure 1 for the means). Less educated participants did not show an effect on candidate education (*b* = −.016). Interestingly, the political orientation of the candidate affected feelings of shared identity for the less educated, but not for higher educated participants (see Supplemental for analyses). In sum, the higher educated feel a shared identity with higher educated candidates, and the lower educated identify with candidates with more conservative orientations.

#### Educational identification

**Figure 2.**
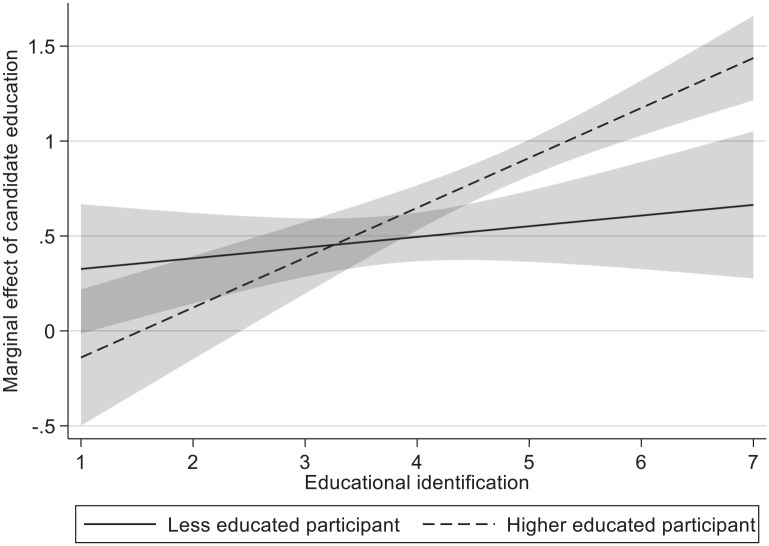
Marginal effect of candidate education on competence for higher and less educated participants across educational identification. *Note.* Marginal effect of candidate education denotes competence of higher educated candidate minus competence of less educated candidate. Shaded areas denote 95% confidence intervals. Y and x-axis values are unstandardized values.

We also added a measure for educational identification (*M* = 4.40, *SD* = 1.08; *M*_le_ = 3.78, *M*_he_ = 4.79). Hence, we investigate whether the interaction between participant education and candidate education is further moderated by educational identification. If educational level of the participant moderates the effect of candidate education, it is likely that these effects are moderated by how strongly participants identify with their educational level. For both competence (*b* = .208, *p* = .006) and vote intention (*b* = .144, *p* = .048), we find significant three-way interactions. Figure 2 plots this three-way interaction effect for competence. We find a significant interaction effect of candidate education and educational identification for higher educated participants (*b*_competence_ = .265, *b*_vote intention_ = .228; both *p*s < .001), which explains the significant three-way effects. In line with our expectations, simple effects of candidate education (i.e., the extent to which higher educated candidates are preferred over less educated candidates) are higher for high identifying (1 *SD* above the mean) higher educated respondents (*b*_competence_ = .485, *b*_vote intention_ = .276; both *p*s < .001), than for low identifying (1 *SD* below the mean) higher educated participants (*b*_competence_ = .220, *p* < .001; *b*_vote intention_ = .049, *p* = .199). As such, the extent to which higher educated participants are more likely to evaluate higher educated candidates more positively or prefer to vote for them thus entirely depends on the extent to which these participants identify with their education. Contrary to our expectations, there are no significant two-way interaction effects of candidate education and educational identification among less educated participants for any of the stereotypes or vote intention. Furthermore, there was a significant interaction with educational identification and feeling of shared identity (*b* = .262, *p* < .001), but this was only significant for the higher educated (Candidate Education × Educational Identification Effects: *b*_LE_ = −.022, *p* = .719; *b*_HE_ = .241, *p* < .001).

#### Vote intention mediated by competence

How does competence relate to vote intention? The results from the regression analyses, which includes all stereotype dimensions, show that competence is significantly related to vote choice (*b*_competence_ = .130, *p* < .001) and is moderated by participant education (*b* = .103, *p* = .016) such that the effect is stronger for higher educated participants: The simple effect for the higher educated is .160 (*M*_low competence_ = 3.74, *M*_high competence_ = 4.28, *p* < .001), and for the less educated this is .057 (*M*_low competence_ = 3.85, *M*_high competence_ = 4.04, *p* < .001). Identification with the candidate is also significantly related to vote intention (*b* = .426, *p* < .001), and this is stronger for higher than for less educated (*b* = .173; simple effects: *b*_LE_ = .320, *b*_HE_ = .493; all *p*s < .001).

We calculate the indirect effects by using the sample estimates to generate a distribution of both the *a* and the *b* coefficients (in standard mediation terms) and standard errors, and then calculate the product of these two distributions, and the 95% CI as the estimation of the indirect effect (Monte Carlo method; MacKinnon et al., 2004). Looking at these indirect effects of candidate education through the stereotypes, competence has a significant and substantial indirect effect (*b* = .069, 95% CI = [.052, .087]). There is also a small indirect effect through morality (*b* = .006, 95% CI = [.002, .010]).

### Discussion

Despite mixed evidence in previous research, we find a strong preference for higher educated political candidates. In line with institutional theories of the educational system, the effect of candidate education on voting preferences is explained by the higher perceived competence of higher educated candidates. According to these theories, higher education plays an important part in cultivating the belief in meritocracy, where individuals should be mainly judged on their (individual) competence. This centrality of competence is reflected in our results.

Educational identification moderates the preferences of higher educated: while both low and high identifying higher educated are positive about higher educated candidates, high identifying higher educated are more positive. This suggests that ingroup bias motivations play a role for the higher educated, as highly identified group members show more ingroup bias (Voci, 2006).

The higher competence perception and vote intention for higher educated candidates are significantly weaker among the less educated participants: They are less negative about less educated candidates than higher educated participants and, when it comes to vote intentions, less positive about higher educated candidates. All these effects are found while controlling for political orientations of the participants. In the introduction we explained that for less educated participants there are two opposing effects: Favoring a competent candidate could lead to a preference for a higher educated candidate but ingroup bias could lead to a preference for a less educated candidate. They do perceive the higher educated candidates as more competent than less educated candidates, but they do not *identify* more strongly with higher compared with less educated candidates. The stronger voting intention for higher educated candidates seems to be driven by perceived competence, but something (presumably education group membership as they are not a member of this group) prevents them from really identifying with these candidates. In any case, moderation of voting preferences by identification was absent for the less educated, which might imply that identity and ingroup bias is less likely to play a role in their preferences toward candidates.

This raises a new question: If perceived competence mediates the effect of candidate education, would people still prefer higher educated candidates even if higher and less educated candidates were equally competent? Or do people simply prefer more competent candidates and is the educational level only used as a heuristic for estimating a candidate’s competence? Answering this question will tell us whether education or competence per se is the critical predictor. This is what we set out to test in Study 2, where we simultaneously independently manipulate candidate education and candidate competence.

## Study 2

Study 1 revealed the strong relevance of competence in explaining the preference for higher educated political candidates. Does this preference still exist when we (independently) manipulate the competence of the candidates we present to the participants? Thus, in our profiles of the candidates, we manipulated education, as in Study 1, and also the level of competence per se. We changed the stereotype characteristics that we ask the participants to rate the candidates on to focus on (different aspects of) competence. This allows us to assess on which aspects of competence participants see the largest difference between higher and less educated candidates.

### Method

In Study 2, we again use a Dutch nationally representative sample, but we changed the experimental setup slightly. We based our sample size on the statistical power analysis in Study 1. Method and measures are similar to Study 1, except when mentioned below. Results from reliability analyses can be found in Supplemental Appendix 8. We use the same analytical strategy outlined in Study 1.

#### Participants

The sample had an initial size of 1,367 participants who completed the questionnaire. Of these, 157 were removed for still being in education. Then we removed one respondent who was younger than 18. We also removed 393 individuals who did not pass the attention check question.^9^ The final sample consists of 816 participants (413 men, 402 women, one “other,” mean age = 53.1, *SD* = 14.4). Sensitivity analyses with the same assumptions as the power analysis in Study 1, with 816 participants (correlations among repeated measurements in this study = .3), give a minimum effect size of *f* = .054.

#### Candidate educational level

In the profiles, we again use a manipulation of educational level. Our higher educated candidate is still master’s level, but our less educated is now someone who did not follow any education after s/he was 16 (17 in the alternative text). The previous manipulation could be interpreted as someone who finished the academic track in high school, which is, in the Dutch context, often seen as more middle than less educated. Although this does not necessarily mean that participants saw the candidate as “middle” educated, we changed the text to something that is more unambiguously less educated. The text of the manipulation can be found in Supplemental Appendix 3.

#### Candidate competence

Our goal is to assess whether manipulating the competence of the candidate neutralizes the effect of the education of the candidate. To maintain the realism of our manipulation, and to avoid focusing on one particular aspect of competence, we opted to refer to the candidate’s (successful) experience in the political field. As we are not interested in any specific form of competence, we opted for a naturalistic manipulation that provided (implicitly) information on domain-relevant knowledge. Although experience is not necessarily competence per se, and our study does not allow us to distinguish between these two, it signals that this person is likely to have domain-relevant knowledge and skills. To increase the strength, we gave the “competent” candidates experience of more than one term, to indicate that this candidate has been re-elected. The noncompetent candidate is presented as someone who does not have any previous political experience. The competent candidate is presented as someone who was a councilor in the local government of a medium-sized municipality. To increase the visibility of this experience, we mentioned two portfolios the candidate was responsible for and previous experience before going into politics (all relatively neutral factors). Example manipulation text (competent): “[name] is, since the municipal elections of 2014, councilor in a medium-sized municipality. S/he has among others, traffic and transportation in his/her portfolio. Before s/he entered politics s/he rose through the ranks as manager in the municipality.” See Supplemental Appendix 3 for the text of all manipulations.

#### Candidate perceptions

We also changed the stereotype candidates were rated on. We have now an expanded list of competence related traits, that can be grouped into five different dimensions of competence: practical competence (example item: “hard working”; *r* = .762), theoretical competence (example item: “intelligent”; *r* = .848), rhetorical competence (example item: “linguistically proficient’; *r* = .773), social competence (example item: “empathic”; *r* = .603), strategic competence (example item: “tactical”; *r* = .642). This is not an exhaustive list, but refers to five dimensions of competence that are often seen as relevant in the political realm, and where higher and less educated individuals might diverge in their preferences. Theoretical competence comes closest to the competence measure from Study 1, as they share one trait (“intelligent”). We also asked the participants to rate the candidates on commitment and morality (both with one item). We list all traits in Supplemental Appendix 5. In the “Results” section, we primarily use a composite scale of all dimensions of competence (*composite competence scale*), based on the mean of the five dimensions (α = .917).

#### Procedure

Study 2 followed the same procedure as Study 1.

### Results

As in Study 1, we built the models stepwise; see Supplemental Appendix 9 for a detailed overview of our models.

#### Main effects

Manipulations of candidate education and candidate competence had main and interaction effects on perceived competence. In Table 1, the main effects are presented for the composite competence scale and also for all subdimensions of competence. For overall competence, we find positive effects of candidate education and candidate competence, with the former being slightly larger. In addition, we find an interaction between participant education and candidate education (*b* = .106, *p* = .002), but not for participant education and candidate competence (*b* = .031, *p* = .236). Both higher and less educated see higher educated candidates as more competent, but this relationship is again significantly stronger for the higher educated (*b*_LE_ = .211, *b*_HE_ = .317; all *p*s < .001). There was also an interaction between candidate education and candidate competence, −.179 (*p* < .001). The effect on perceived competence of being higher educated or being competent is strongest when the candidate is less competent or less educated, respectively (simple effects of education: *b*_noncompetent_ = .381, *b*_competent_ = .202; simple effects of competence: *b*_low educated_ = .327, *b*_high educated_ = .148; all *p*s < .001).

#### Educational identification

**Figure 3.**
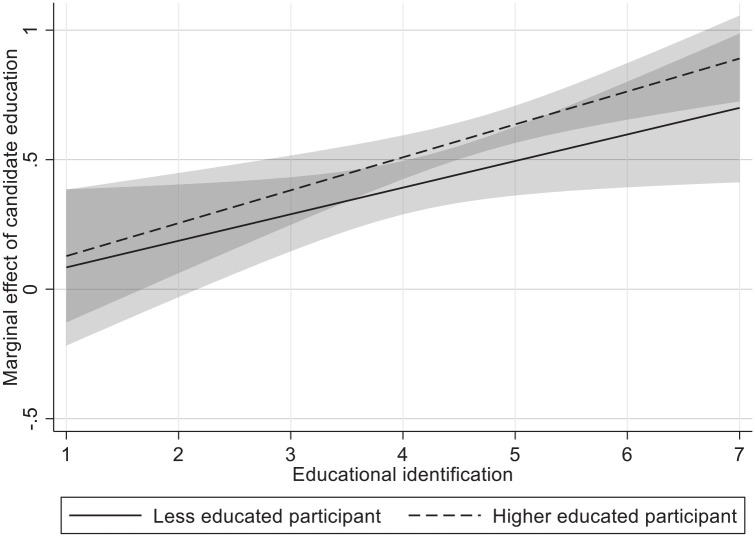
Marginal effect of candidate education on competence for higher and less educated across educational identification. *Note.* Marginal effect of candidate education denotes competence of higher educated candidate minus competence of less educated candidate. Shaded areas denote 95% confidence intervals. Y and x-axis values are unstandardized values.

In the next model, we add educational identification (*M* = 4.49, *SD* = 1.07, *M*_le_ = 4.09, *M*_he_ = 4.72) and its interaction terms in a three-way interaction with participant and candidate education. In contrast to Study 1, there is no significant three-way interaction between candidate education, participant education, and their educational identification on competence (*b* = .027; *p* = .672), as both partial interactions for higher and less educated participants are (similarly) significant (*b*_LE_ = .115, *p* = .029; *b*_HE_ = .143, *p* < .001). As such, we do find a similar (partial) interaction for higher educated as in Study 1, although here identification also positively moderates the preference of the less educated participants. Hence, the three-way interaction is unlikely to yield a significant result (unlike Study 1), despite the similar role of identification for the higher educated (like Study 1). We have illustrated this in Figure 3.

#### Vote intention

Participants are more likely to vote for higher educated and more competent political candidates (*b*_candidate education_ = .203, *p* < .001; *b*_candidate competence_ = .239, *p* < .001). Both relationships are moderated by participant education (*b*_candidate education_ = .244, *p* < .001; *b*_candidate competence_ = .084, *p* = .027). Looking at the simple effects of candidate education for less and higher educated, we find that, while the higher educated strongly prefer higher educated candidates (*b* = .303, *p* < .001), less educated do not significantly prefer higher educated candidates (*b* = .059, *p* = .090). Whereas in Study 1, less educated still preferred higher over less educated candidates; when competence is also manipulated here, this preference is only minimal and nonsignificant. The effect of candidate competence is also smaller for less educated respondents compared with higher educated respondents, but it remains strong and significant for the less educated (*b* = .211, *p* < .001). It, thus, seems that the less educated prefer higher educated candidates for their higher perceived competence (Study 1) and largely ignore information on educational level when information on competence is provided (Study 2). Crucially, higher educated participants prefer higher educated candidates over less educated candidates by a large margin (*b* = .303, *p* < .001) while *at the same time* also preferring more competent candidates over less competent candidates (*b* = 0.295, *p* < .001).

**Figure 4.**
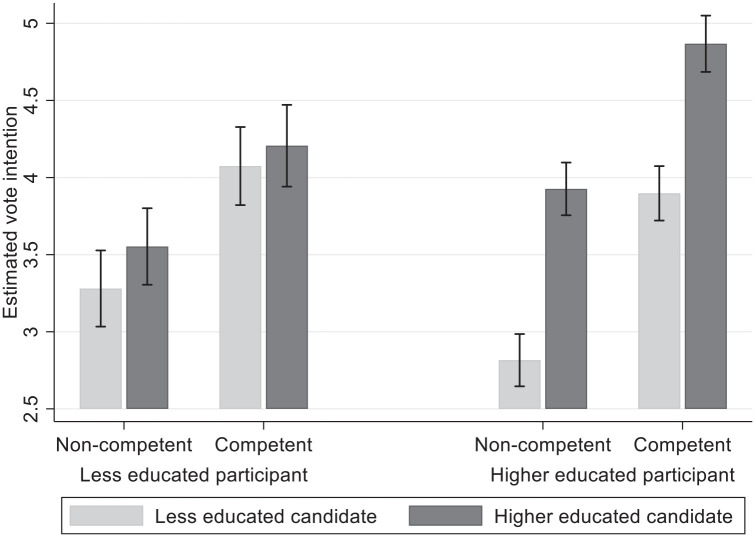
Vote intention scores for all four profiles, for less and higher educated participants. *Note.* Error bars denote 95% confidence intervals. Y-axis values are unstandardized values.

We illustrate this in Figure 4. This figure shows the (unstandardized) scores of the four profiles on vote intention for less educated and higher educated participants. Although the less educated still show a (nonsignificant) preference for higher educated candidates over less educated ones, they significantly prefer a competent less educated candidate over a noncompetent higher educated candidate (the middle two bars for less educated participants; *b* = .152, *b*_unstandardized_ = .522, *p* < .001). The higher educated do not make a distinction between noncompetent higher educated and competent less educated candidates (*b* = −.008, *b*_unstandardized_ = −.029, *p* = .784). This shows not only that the less educated mainly take the manipulated competence as a basis for their vote intention, but that the higher educated take the educational level of the candidate as a basis for their vote intentions, above and beyond the (manipulated) competence of political candidates. For the higher educated, this is not consistent with an explanation based on the academic competence ideology alone, but suggests that ingroup bias also plays a role.

**Figure 5.**
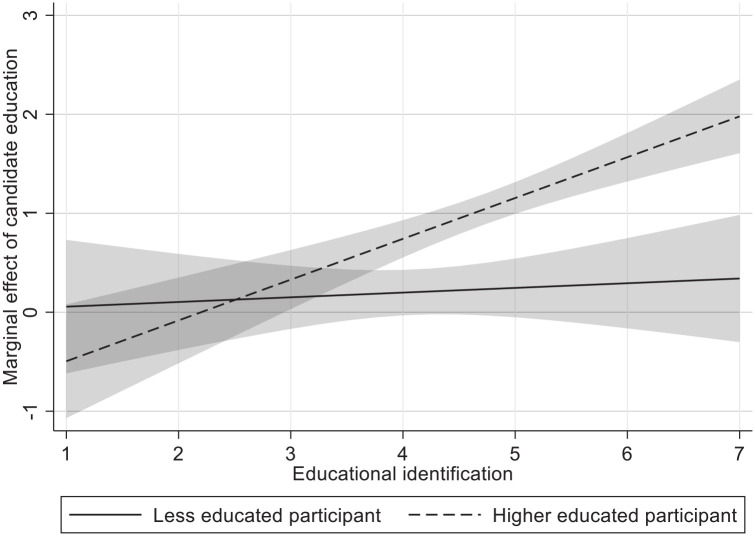
Marginal effect of candidate education on vote intention for higher and less educated across educational identification. *Note.* Marginal effect of candidate education denotes competence of higher educated candidate minus competence of less educated candidate. Shaded areas denote 95% confidence intervals. Y and x-axis values are unstandardized values.

We find a significant three-way interaction with educational identification (*b* = .227, *p* = .005). This interaction is shown in Figure 5. Although identification plays no role in the vote intention for the less educated, higher educated prefer higher educated candidates more when their educational identification is stronger (*b*_HE_ = .256, *b*_low id_ = .146, *b*_high id_ = .404; all *p*s < .001). The means indicate that the positive effect of candidate education among strongly identifying higher educated participants is entirely due to a stronger vote intention toward higher educated (less educated candidate: *M*_low id_ = 3.325, *M*_high id_ = 3.332; higher educated candidate: *M*_low id_ = 3.830, *M*_high id_ = 4.716). In fact, Figure 5 shows that the preference of higher educated participants for higher educated candidates is entirely dependent on identification with educational level. This corroborates that higher educated candidates are motivated by ingroup bias, and this leads them to favor higher educated candidates, above and beyond the competence of the candidate.

How are these vote intentions affected by the different aspects of perceived competence? All five aspects of competence are significantly related to vote intention (all entered in the same model), although the strongest relationships can be found with practical and theoretical competence (*b*_practical_ = .144, *p* < .001; *b*_theoretical_ = .160, *p* < .001; *b*_rhetorical_ = .060, *p* = .023; *b*_social_ = .056, *p* = .006; *b*_strategic_ = .064, *p* = .009). The relationships of theoretical, rhetorical, and strategic are moderated by participant education (*b*_theoretical_ = .206, *p* < .001; *b*_rhetorical_ = .133, *p* = .003; *b*_strategic_ = .093, *p* = .033), that is, higher educated participants weigh their perception of these forms of competence more heavily in their vote intentions than less educated participants do.

All of these aspects also mediate the effect of candidate education on vote intention (see Table 2). Of these, theoretical competence is the strongest mediator. This is in line with the emphasis in educational systems on cognitive ability, closely related to theoretical competence. In Table 2, the coefficients denote the indirect effect. The total effect of candidate education on vote intention is (as reported above) .203 (*p* < .001). Hence, practical mediates around 12% and theoretical competence 34% of the total effect.

**Table 2.** Indirect Effects of Candidate Education, Through Different Aspects of Competence Stereotype, on Vote Intention.

Mediator	Indirect effect	Lower bound	Upper bound
Practical	.024	.015	.034
Theoretical	.070	.046	.095
Rhetorical	.022	.003	.041
Social	.003	.001	.006
Strategic	.013	.003	.023

*Note.* Lower bound and upper bound refer to the bounds of the 95% confidence interval.

### Discussion

In Study 2, we delved deeper into the role of competence as a mediator for the effect of candidate education on vote intentions. We did this in two ways: first, we manipulated not just candidate education, but also candidate competence and second, we expanded our list of stereotype traits associated with competence to five different aspects of competence.

Higher educated candidates are again seen as more competent, and receive higher vote intentions from the participants. However, we find that candidate education is not significantly related to vote intention for less educated participants, though their vote intentions are strongly related to candidate competence. It, thus, seems that the preference for higher educated candidates found in Study 1 among less educated participants disappears when we experimentally control for competence. This is, however, not the case for the higher educated. They prefer competent candidates over noncompetent politicians, but at the same time, they still prefer higher educated over less educated, independent of competence. This suggests that higher educated are motivated by group concerns, seeing higher educated candidates as representatives of “their own group.” This is confirmed by an analysis with educational identification as a moderator, where the preference for higher educated candidates depends entirely on identification for higher educated participants (replicating a key result from Study 1). Further investigation showed that this preference is entirely due to a more favorable assessment of higher educated candidates, an example of ingroup love (Brewer, 1999). Thus, whereas less educated prefer higher educated candidates due to their higher perceived competence, in line with an academic competence ideology related to meritocracy, higher educated are also motivated by ingroup bias.

Interestingly, we find that the effect of candidate education on vote intention is most strongly mediated by *theoretical* competence (e.g., “smart” and “intelligent”). Modern educational systems are, in comparison with older, “classic,” educational systems, marked by a strong emphasis on cognitive ability (Baker, 2014). We do find that the relationship of candidate education with competence is strongest for theoretical competence, corroborating that higher educated are seen mostly as excelling in cognitive skills. Our findings suggest that this specific type of competence is also a forceful factor in electing politicians.

## General Discussion

Despite evidence of a rise in anti-elite parties claiming to represent the “common people,” and evidence of a general education-based political conflict, there is scant public resistance to the fact that the political sphere, in many ways, is dominated by the higher educated. It is in the light of this (apparent) paradox that we investigate how people evaluate higher and less educated candidates and whether they express any preference toward either. Although previous research into this matter has shown conflicting evidence, we demonstrate here across two studies that people generally prefer higher educated candidates over less educated candidates, and this preference seems to be borne mostly out of their perceived higher competence. However, when candidate competence was also manipulated, candidate education no longer affected the preferences of less educated participants. Although higher educated participants prefer competent candidates over less competent candidates, at the same time, they also preferred higher educated candidates over less educated candidates, which suggests that this is due to *ingroup bias*: They prefer higher educated candidates over and above their competence. To the extent that people prefer higher educated candidates due to their competence, this is mostly due to their *theoretical* (cognitive) competence. As such, while political conflict consistently finds opposition between less and higher educated, voter preferences of both less and higher educated still mostly point favorably toward the higher educated. These findings add up to three main conclusions.

Our first conclusion points initially toward a strong acquiescence of less educated people to the dominant position of the higher educated, and their deference toward the higher educated when it comes to political vote choice. Less educated people show a similar pattern as the higher educated toward candidates of different educational levels: They see higher educated candidates as more competent. However, it is worth mentioning that less educated participants did not identify more strongly with higher compared with less educated candidates. Indeed, in contrast to the higher educated any such identification would necessarily be vicarious rather than grounded in the ingroup. In general, the less educated, thus, seem to be restricted in showing ingroup bias, possibly due to the strong reality constraints that are imposed on people by a meritocratic culture and the near uncontested source of competence that education is to the higher educated (Baker, 2014). This results initially, among less educated participants, in generally positive assessments of the competence of higher educated candidates, and people’s vote intentions toward them. However, in Study 2, when we manipulated the competence of the candidates independently, these reality constraints are relaxed, outgroup bias disappears, and it seems that motivated reasoning comes into play (Doosje et al., 1995; Kunda, 1990). Note that by this we do not mean that the less educated are motivationally biased and the higher educated are not. On the contrary, the less educated seem motivated to distinguish competence from educational level when able to do so and to base their judgments on competence per se, whereas the higher educated are not sensitive to this, presumably because it suits their group interests (Packer, 2008). Both of these observations are examples of motivated reasoning (Doosje et al., 1995; Kunda, 1990), but the less educated are arguably more accurate and less biased because motivation based on group interests led to more thorough scrutiny of the education/competence distinction than it did for the higher educated.

This resistance by people with lower levels of education is not only evident from Study 2 where they had the clearest opportunity to distinguish competence and education. The less educated also perceived, in Study 1, a smaller difference in competence between higher and less educated candidates, than did higher educated participants. To put matters in perspective, however, it also remains true that, without experimental manipulations of competence, higher educated candidates were consistently seen as more competent by less educated participants. In other words, resistance among the less educated is subtle and not always present. Moreover, previous research has shown that identification among the less educated is generally lower due to the negative stigma attached to “less educated.” Hence, strong resistance and an education-based *open* conflict are rather unlikely (Jackman, 1994; Spruyt, 2014). Indeed, education seems to be predominantly a basis for group behavior for the higher educated—who identified significantly more with their own group than the less educated.

Our second conclusion is that the dominance of the higher educated as political representatives seems to be based on the assumed competence associated with education, but also on ingroup bias among the higher educated. Across both studies, the higher educated showed stronger ingroup bias when they identified strongly with their educational level, at least in their vote intention and when assessing candidate competence. This suggests that the higher educated are motivated by group concerns related to their educational group membership. They protect their group identity or group interests by favoring ingroup members (Brewer, 1999). This serves as a likely explanation for the almost extreme dominance of higher educated in modern politics (Bovens & Wille, 2017), although a large part of this dominance is not created at the voting booth, but rather through in-party processes such as selection of candidates by party committees and so on (Bovens & Wille, 2017).

Third, education is not merely related to general competence, but to a theoretical or cognitive competence more specifically. In our findings, competence is the most important factor in deciding vote choice, and of the different aspects of competence that we looked at, it is theoretical competence (or cognitive competence) that is most decisive in people’s vote choice. This is reflected in what recent literature on the changes in western educational systems have pointed to: an increasing focus on cognitive ability as the core defining aspect of educational achievement (Baker, 2011, 2014) In the Stereotype Content Model (Fiske, 2018; Fiske et al., 2002), competence is related to status in general. However, our findings indicate that competence is different from cognitive ability. Although theoretical competence was related the strongest to candidate education, practical competence was most strongly related to our candidate competence manipulation. To what extent is educational competence different from how we usually conceptualize competence? Do countries in which education is a more important institution (what Baker, 2014, calls “schooled societies”) have a different conceptualization of competence compared with countries where education is a less central institution? These are interesting avenues for future research.

The current research can also be expanded beyond education to look at combinations or intersections of identities. One way in which this can be understood is to see the distinction between higher and less educated as a distinction of dominant versus nondominant (Jackman, 1994) or in terms of the status of groups. Previous research has shown that stereotypes follow status distinctions such that higher status groups are seen as more competent (Kervyn et al., 2010). In this way, education can intersect with, for instance, gender, where combinations of these identities could be seen as more or less competent or produce relatively unique competence stereotypes. Future research could also investigate these preferences regarding actual rather than fictional politicians (see Callaghan et al., 2018).

Overall, this research tells a story of how higher educated candidates are seen as more “electable,” but also of how the higher educated *as a group* defend their interests (consciously or not), whereas the less educated are prevented from doing so. The combination of (a) a higher perceived competence, that is mainly seen as a cognitive advantage over the less educated, and which (b) is seen as the most important factor for electability, and (c) an ingroup bias among higher educated whereby (strongly identifying) higher educated favor higher educated regardless of competence, and (d) an absence of ingroup bias among the less educated due to an assumption of higher competence of the higher educated, contributes to an almost unavoidable dominance of higher educated in modern politics. *Almost* unavoidable, that is. There is a silver lining for the less educated. When given the chance, the less educated do not simply show deference to the higher educated, but, unlike the higher educated themselves, accord more weight to candidate competence over education per se. As such, deference should not necessarily be taken as proof of political preference.

## Supplemental Material

sj-docx-1-psp-10.1177_01461672221077794 – Supplemental material for When and Why People Prefer Higher Educated Politicians: Ingroup Bias, Deference, and Resistance Click here for additional data file. Supplemental material, sj-docx-1-psp-10.1177_01461672221077794 for When and Why People Prefer Higher Educated Politicians: Ingroup Bias, Deference, and Resistance by Jochem van Noord, Toon Kuppens, Bram Spruyt and Russell Spears in Personality and Social Psychology Bulletin

## Notes

1. Throughout the article, we use the terms “less” and “higher” educated. This terminology is part of popular and official discourse in the Netherlands (where our study is situated). Although it corresponds to the educational level someone has attained, it does not reflect a value judgment about any type of formal education. Specifically, with less educated, we include those without higher secondary education, and higher educated those with a bachelor’s degree or higher.

2. Including in the Netherlands, where our study is situated.

3. We also did a pilot study on a student population. See Supplemental Material (Supplemental Appendix 1) for more details and results.

4. These are only limitedly discussed in the “Results” section; full results on these stereotypes can be found in the Supplemental Material.

5. No studies in this manuscript were preregistered. Materials and data are available at https://osf.io/2uc4n/?view_only=6e560e7403a4494c9a53e8524e0f29c5

6. Analyses that did include these participants showed mostly slightly weaker relationships, but no substantive differences nor differences in significance.

7. One half had the education question at the end of the survey as manipulation of educational salience. Details and results are discussed in Supplemental Appendix 11.

8. As dependent variables are measured on a .5 *SD* scale, this translates into 2 × .282 = .564 *SD* difference between higher and less educated candidates.

9. Analyses that did include these participants showed mostly slightly weaker relationships, but no substantive differences nor differences in significance.
