# Supplementary material for: When and Why People Prefer Higher Educated Politicians: Ingroup Bias, Deference, and Resistance
Source: Pers Soc Psychol Bull. 2022 Feb 22;49(4):585–99. doi: 10.1177/01461672221077794 (PMC9989228; doi:10.1177/01461672221077794)
Supplement: sj-docx-1-psp-10.1177_01461672221077794 – Supplemental material for When and Why People Prefer Higher Educated Politicians: Ingroup Bias, Deference, and Resistance [file sj-docx-1-psp-10.1177_01461672221077794.docx]

**Appendix**

## **Appendix 1: pilot study**

We collected data on two samples among the same population (bachelor students). As the surveys were identical and results similar (results for the two separate samples are available upon request), we present these results as one sample. The survey is, apart from the subjective education manipulation, identical to Study 1 in the main text, we refer to the method section of the main text for the full operationalization of all other measures and the survey procedure.

## **Method**

Participants rated four fictitious political candidates who varied in educational level and political preference. We manipulated the scale of the question about respondents’ educational level to lead them to perceive their actual educational level (beginning bachelor students) as lower or higher than others (see below for more information). We refer to this manipulation as ‘subjective education’. Gender of the political candidates was matched to participant gender. This study therefore had a 2 (subjective education: high versus low) by 2 (candidate education: high versus low) by 2 (candidate political preference: progressive versus conservative) design, with the last two factors varying within participants.

***Participants***

We collected data on two samples of bachelor students. In these two samples, 84 and 156 first year students at a Dutch university participated in exchange for a small payment (sample 1) or course credits (sample 2). The first sample also included non-bachelor students, but we selected only the bachelor students for analyses because of the manipulation of subjective educational level (see below). We excluded eleven participants for not passing the attention check question (‘Choose agree strongly if you have read this question’). Both samples only include students with a Dutch nationality, though we have no information on ethnic background beyond nationality. The final, combined, sample consists of 229 participants (166 women, 61 men, 1 other, 1 gender unknown; mean age = 22.1, SD = 5.35).

***Subjective educational level manipulation***

In both studies we manipulated students’ subjective educational level. We asked students to indicate their educational level in a manipulated education question. One half (46.7%) were asked about their current *attained* level, which for first year bachelor students is high school. Due to the selection of answer categories, this is the lowest category of education (*secondary education* / bachelor / master / doctor). The other respondents were asked about the educational level they were currently following, which in the answer categories we provided is the highest level (secondary education / vocational tertiary / bachelor at applied sciences / *university*). Hence, the first group is coded as ‘low subjective education’ and the second as ‘high subjective education’.

***Candidate perceptions***

We asked participants to rate four political candidates on a total of eight characteristics (two per stereotype dimension) associated with competence (correlation between the two items: r = 0.68,), warmth (r = 0.78), agency (r = 0.59), and morality (r = 0.64) and their willingness to vote for each of these candidates. Additionally, we measured with two items (r = 0.85, example item: “to what extent do you think you can identify with this candidate?”) to what extent the participants identified with the presented candidates (*feeling of shared identity)*. See Appendix 5 for all stereotypes and shared identity items.

***Educational identification***

We measure identification with ten items (α = 0.88, items are listed in Appendix 6) adapted from Leach et al. (2008).

***Control variables***

We include age and gender as control variables. We also include three variables on political preferences: environmentalism (four items, α = 0.74), law and order (four items, α = 0.58), and ethnic prejudice (four items, α = 0.88). We listed all items in Appendix 7.

## **Results**

We start with models containing only main effects and the interaction between candidate education and candidate political orientation (and control variables), with the different stereotypes as the dependent variables.

***Main effects and moderation by subjective education***

Higher educated candidates are seen as more competent (M_LE_ = 4.00, M_HE_ = 5.56, b = 0.625, p < 0.001), more agentic (M_LE_ = 4.67, M_HE_ = 5.16, b = 0.235, p < 0.001), and more moral (M_LE_ = 4.82, M_HE_ = 5.10, b = 0.142, p = 0.002) than less educated candidates. Participants did not make a distinction between higher and less educated on warmth (M_LE_ = 4.96, M_HE_ = 5.02, b = 0.027, p = 0.320). Participants were more likely to indicate that they would vote for higher educated candidates (M_LE_ = 4.02, M_HE_ = 5.68, b = 0.331, p < 0.001). Additionally, participants were more likely to feel a shared identity with higher educated candidates (b = 0.287, p < 0.001). Now, we add to this model the two interactions between participants’ subjective educational level and candidate education and political orientation. We did not find any significant interactions between participants’ subjective and candidates’ educational level (all ps > .10, except shared identity p = 0.089). Main effects of subjective educational level are all small and non-significant (all bs < 0.033, all ps > 0.3).

***Educational identification***

Next, we added educational identification, its interaction term with candidate education and subjective education, and the three-way interaction term between candidate education, participant subjective education, and educational identification to our first model, to explore how educational identification moderates the attitudes of our participants. Table 1 summarizes the coefficients of this three-way interaction (including a breakdown into two-way interactions and the simple effects). Results show a significant, negative, three-way interaction effect for agency and morality. These effects indicate that all groups have more positive perceptions of the higher educated candidate than the less educated candidate, but that this education effect was absent for those in the low subjective education condition and who identify weakly with their education level (see Table 2, last column). This pattern, for agency, is also depicted in Figure 1. Apparently, people that chronically value education less (low identification) and have been led to believe that their education level is not so high (low subjective education) give much less attention to education in judging political candidates. It thus seems that our manipulation of subjective education can induce the feeling that one ‘is’ less educated, and that this feeling induces more positive assessments of less educated candidates but only among those with a low identification with their (actual) educational level. For this to be the case, we would expect a significant two-way interaction between candidate education and subjective education among those with low identification. This is the case for agency (b = 0.273, p < 0.001), but not significantly so for morality at 1 SD below the mean of identification (b = 0.108, p = 0.106), even though the pattern was similar. As such, this suggests that manipulating (subjective) educational level can have effects, such that for the combination of conditions where educational level was least positive and least valued, the perceived disadvantage of the less educated candidates was reduced, at least for agency. Note that identification is measured with others who have a similar educational level, which refers to respondents’ ‘true’ educational level, rather than their manipulated subjective level. There are no statistically significant differences between participants with high and low subjective education on educational identification (b = 0.096, p = 0.146). While we do not find significant three-way interactions for competence and vote intention, we do see stronger preferences for higher educated candidates when they identify strongly with their (higher) educational level.

**Table 1**

*Summary of the three-way interactions of candidate education, participant subjective education, and educational identification*

|  |  | High subjective education | | | Low subjective education | | |
| --- | --- | --- | --- | --- | --- | --- | --- |
| Dependent variable | Three-way | Two-way | High ID | Low ID | Two-way | High ID | Low ID |
| Competence | -0.044 | 0.168* | 0.741*** | 0.573*** | 0.212** | 0.699*** | 0.487*** |
| Warmth | -0.012 | 0.022 | 0.021 | -0.000 | 0.034 | 0.064 | 0.030 |
| Agency | -0.394*** | -0.094 | 0.233*** | 0.328*** | 0.299*** | 0.354*** | 0.055 |
| Morality | -0.276** | -0.072 | 0.099* | 0.172*** | 0.203** | 0.267*** | -0.064 |
| Shared identity | -0.106 | 0.133 | 0.395*** | 0.262*** | 0.239** | 0.367*** | 0.128* |
| Vote intention | -0.092 | 0.154 | 0.425*** | 0.271*** | 0.246** | 0.443*** | 0.197*** |

*Note.* Three-way refers to the full three-way interaction. Two-way refers to candidate education * participant educational identification interactions. High/low ID refers to the simple effects of candidate education of high/low subjective education participants with high/low educational identification (1 SD above and below the mean of the unstandardized scale). Coefficients denote standardized (with mean = 0, SD = 0.5) coefficients, so a simple effect of 0.5 means that the higher educated candidate scores 1SD higher on the DV then the less educated candidate.

*** p < 0.001, ** p < 0.01, * p < 0.05.

**Figure 1**

*Estimated agency across participant and candidate education, and educational identification (Study 1b)*

*Note.* Error bars denote 95% confidence intervals. Values along the y-axis are the unstandardized values.

***Vote intention mediated by stereotypes***

We also look at whether the stereotypes mediate the effect of candidate education on vote intention. First of all, all stereotypes are related to vote intention, though only the effects of competence, warmth, and shared identity remain significant when they are entered in the model simultaneously (competence: b = 0.213, p < 0.001; warmth: b = 0.126, p < 0.001; shared identity: b = 0.327, p < 0.001). After including all stereotypes, the effect of candidate education decreases from 0.331 to 0.090, which is still significant at p = 0.005. We calculate the indirect effects by using the sample estimates to generate a distribution of both the *a* and the *b* coefficients (in standard mediation terms) and standard errors, and then calculate the product of these two distributions, and the 95% confidence interval as the estimation of the indirect effect (Monte Carlo method; MacKinnon et al., 2004). Looking at these indirect effects of candidate education through the stereotypes, only competence mediates a significant and substantial indirect effect: 0.201 (95% CI = [0.150, 0.255]).

## **Discussion**

Higher educated political candidates are evaluated more positively on competence, agency, and morality, and participants report stronger intentions to vote for higher educated candidates. We also manipulated the subjective perception of participants’ own educational level and measured educational identification. Participants for whom education was chronically less important (i.e., low identification) and who were led to believe that their educational level was not so high (i.e., low subjective education), attached less importance to the educational level of political candidates, when judging their agency and morality. For this subgroup we find some evidence that they questioned the relevance of education, even though the effect was less clear and not significant for competence and vote intention. Our manipulation of subjective education aimed to induce the feeling of being less educated, and for those who also identified weakly with their educational groups, this combination presumably produced the best conditions for downplaying the importance of candidate education.

## **Appendix 2: power analysis Study 1**

There are several focal analyses in Study 1 and we use multilevel analyses that are not easily used in power calculations, but we nevertheless did an approximate power calculation. The effect of candidate education on voting intention had a Cohen’s d effect size of 0.66 in the pilot study (see Supplemental Material), so we took a d of 0.5 as a conservative estimate of the effect size. If we want to have sufficient power to detect whether this is moderated by participant education, and if we assume an attenuated interaction, the standardized effect size could be four times smaller (see Giner-Sorolla, 2018), so d = 0.125 (or f = 0.0625). We base the required sample size on a repeated measures model using *GPower*. For a within-between interaction with Cohen’s f = 0.0625, alpha = 0.05, power (1-ß) = 0.8, three groups (i.e., less, middle, higher educated), four measurements (i.e., candidate profiles), and an average correlation of 0.2 over the measurements (i.e. stereotypes across candidate profiles, based on the pilot study) the required sample size is 702.

## **Appendix 3: manipulation texts**

***Education (all studies, Study 3 has a slightly different text for less educated)***

Less educated

1. [candidate name] has a high school diploma *(Study 3: didn’t follow education after his sixteenth)*, and was, during his/her time in high school active in the organizing of school nights and parties.
2. [candidate name] has a high school diploma *(Study 3: quit education when he was 17)*, and was, during his/her time in high school active in the korfball association

Higher educated

1. [candidate name] has attained his master diploma at the university, and was, during his time at the university active for a study association
2. [candidate name] has attained his master diploma at the university, and was, during his time at the university active for the volleyball club

***Political orientation (Study 1 and 2)***

Progressive

1. [candidate name] dedicates himself to the climate. He wants more money for energy use reductions and waste recycling.
2. [candidate name] thinks that sustainability deserves more attention. He wants us to make a bigger priority of the transition to sustainable energy.

Conservative

1. [candidate name] dedicates himself to a safer society. He thinks there should be more police agents on the street.
2. [candidate name] thinks safety is an important issue. He thinks that criminals should be punished more harshly and without reservations.

***Competence (Study 3)***

Low competence

1. [candidate name] has no prior political experience.
2. [candidate name] is putting himself up for election for the first time

High competence

1. [candidate name] has been a local councillor at a medium-sized municipality for five years. He has, among others, living and land affairs in his portfolio. Before he entered politics he was successful as a creative entrepreneur.
2. [candidate name] has been a local councillor in a provincial city since the municipal elections of 2014. He is mandated for, among others, traffic and transport. Before he entered politics he rose up the ladder as a manager in the municipality.

**Results of analyses with political orientation of the candidate**

*Pilot study*

The manipulation of political orientation revealed that conservative candidates are seen as significantly less competent (b = -0.091, p < 0.001), less warm (b = -0.335, p < 0.001), and less moral (b = -0.110, p = 0.002), but more agentic (b = 0.098, p < 0.001). Conservative candidates also received lower vote intentions from the participants (b = -0.219, p < 0.001) and participants reported weaker feelings of shared identity with conservative candidates (b = -0.174, p < 0.001). None of these results were moderated by participant subjective education, nor in a three-way interaction with candidate political orientation * participant subjective education * educational identification – except shared identity (three-way interaction: b = 0.357, p = 0.005) such that participants with high subjective education report more feelings of shared identity when they identify weakly (b_twoway_ = 0.287, p = 0.003; simple effects: b_low identification_ = -0.374, p < 0.001; b_high identification_ = -0.088, p = 0.161).

*Study 1*

In Study 1 conservative candidates were seen as less warm (b = -0.045, p = 0.002), more agentic (b = 0.067, p < 0.001), and more moral (b = 0.038, p = 0.004). They also received more vote intentions (b = 0.086, p < 0.001). No significant differences for competence (b = -0.001, p = 0.965). Participants also reported stronger feelings of shared identity with conservative candidates (b = 0.101, p < 0.001). All these relations were negatively moderated by participant education (b_competence_ = -0.090, p = 0.012, b_warmth_ = -0.173, p < 0.001, b_agency_ = -0.105, p = 0.003, b_morality_ = -0.116, p = 0.001, b_shared identity_ = -0.186, p < 0.001, b_vote intention_ = -0.209, p < 0.001). Hence, less educated participants were more positive about conservative candidates than higher educated participants. These relationships were not moderated by either educational salience or identification. Hence, political orientation of the candidates affected vote intention and assessment of voters independently from educational level, and vice versa.

## **Appendix 4: Profile text**

Sven/Ilse Verhoeven was born on 23 July 1978 and lives in Noord-Brabant. [Education manipulation.] He/she is married and has two children. In his/her free time he/she listens often to music and likes to go to a concert. Besides that, he/she has been playing the guitar from a young age. [Political orientation/competence manipulation.]

Filip/Laura Kramer was born on 8 November 1977 and lives in Noord-Holland. [Education manipulation.] He/she co-habitates and has two children. In his/her free time, he/she likes to go for a walk, go for a run and travel, and always has a good book to read. [Political orientation/competence manipulation.]

Pim/Julie de Vries was born on 26 January 1979 and lives in Zuid-Holland. [Education manipulation.] He/she is married and expecting a first child. He/she spends his/her free time mainly on photography, spending time with family and friends, and make plenty of time for travelling. [Political orientation/competence manipulation.]

Joeri/Hanne Smit was born on 17 March 1976 and lives in Gelderland. [Education manipulation.] He/she is married and has a child. In his/her free time she tries to go for a run a couple of times a week and likes to go out for dinner with friends. [Political orientation/competence manipulation.]

**Appendix 5**

**Table A1**

*Stereotype characteristics and dimensions*

| Study 1 & study 2 | | Study 3 | |
| --- | --- | --- | --- |
| Dimension | Characteristic | Dimension | Characteristic |
| Competence | Capable [Bekwaam] | Practical competence | Decisive [Daadkrachtig] |
|  | Intelligent [Intelligent] |  | Hard working [Hardwerkend] |
| Warmth | Friendly [Vriendelijk] | Theoretical competence | Intelligent [Intelligent] |
|  | Sympathetic [Sympathiek] |  | Smart [Slim] |
| Agency | Decisive [Daadkrachtig] | Rhetorical competence | Eloquent [Welbespraakt] |
|  | Confident [Zelfverzekerd] |  | Linguistically proficient [Taalvaardig] |
| Morality | Honest [Eerlijk] | Social competence | Empathic [Empathisch] |
|  | Trustworthy [Betrouwbaar] |  | Social [Sociaal] |
|  |  | Strategic competence | Tactical [Tactisch] |
|  |  |  | Competitive [Competitief] |

*Note.* Original Dutch term in brackets

Items for shared identity with the candidate:

To what extent do you think that ..?

1. You can identify yourself with this candidate?
2. You can recognize yourself in this candidate?

**Results of analyses with stereotypes as dependent variables**

For the Pilot study see Appendix 1.

*Study 1*

Higher educated candidates were perceived as more competent (b = 0.282, p < 0.001), more agentic (b = 0.099, p < 0.001), and more moral (b = 0.038, p < 0.001), but not more warm (b = 0.002, p = 0.848). This was moderated by participant education for only competence (b = 0.175, p < 0.001), and not for warmth (b = 0.016, p = 0.595), agency (b = 0.023, p = 0.480), and morality (b = 0.033, p = 0.270). Three-way interactions (candidate education * participant education * educational identification) were significant for competence (see main text), warmth (b = 0.129, p = 0.034), and morality (b = 0.145, p = 0.013), and not agency (b = 0.054, p = 0.406). These three-way interactions are such that there is a significant two-way interaction for higher educated participants (b_warmth_ = 0.135, p < 0.001; b_morality_ = 0.160, p < 0.001), where high identifying higher educated perceive differences in stereotypes (b_warmth_ = 0.058, p = 0.008; b_morality_ = 0.118, p < 0.001), but not low identifying higher educated participants (b_warmth_ = -0.076, p = 0.016; b_morality_ = -0.041, p = 0.176). Low identifying higher educated participants even perceive *less* educated candidates as being significantly more warm.

**Appendix 6: scale items of educational identification**

1. I feel a bond with people with my educational level.
2. I feel solidarity with people with my educational level.
3. I feel committed to people with my educational level.
4. I am glad with my educational level
5. I think that people with my educational level have a lot to be proud of.
6. It is pleasant to have my educational level.
7. My educational level gives me a good feeling.
8. I often think about my educational level.
9. My educational level is an important part of my identity.
10. My educational level is an important part of how I see myself.

Adapted from Leach et al. (2008)

**Appendix 7: scale items of the political attitudes**

Ethnic prejudice

1. People with a migration background are usually not to be trusted.
2. People with a migration background form a threat to our culture and customs.
3. The presence of different cultures is a threat to our society.
4. People with a migration background make the crime problems in this country worse.

Law and order

1. People that commit crimes should receive harsher punishments.
2. All laws should be enforced strictly.
3. You should never break the law, even if you would do something right.
4. The death penalty should be introduced for serious crimes.

Environmentalism/attitude towards climate change

1. Climate change is mainly caused by human activity.
2. People worry too much about societal progress and not enough about climate.
3. Climate problems cannot be solved without big changes in our way of living.
4. Almost everything we do in modern life harms nature.

# Appendix 8

**Table A2**

*Results from reliability analyses and correlations*

|  | Pilot study a | Pilot study b | Study 1 | Study 2 |
| --- | --- | --- | --- | --- |
| Reliability analysis (Cronbach's alpha) | |  |  |  |
| Educational identification | 0.88 | 0.87 | 0.90 | 0.90 |
| Ethnic prejudice | 0.89 | 0.87 | 0.94 | 0.93 |
| Law and order | 0.65 | 0.57 | 0.64 |  |
| Environmentalism | 0.73 | 0.73 | 0.81 |  |
|  |  |  |  |  |
| Correlations (Pearson's r) |  |  |  |  |
| Competence | 0.68 | 0.67 | 0.77 |  |
| Warmth | 0.79 | 0.77 | 0.81 |  |
| Agency | 0.55 | 0.61 | 0.76 |  |
| Morality | 0.64 | 0.65 | 0.80 |  |
| Shared identity | 0.88 | 0.83 | 0.89 |  |
| Practical competence |  |  |  | 0.76 |
| Theoretical competence |  |  |  | 0.85 |
| Rhetorical competence |  |  |  | 0.77 |
| Social competence |  |  |  | 0.60 |
| Strategic competence |  |  |  | 0.64 |

**Appendix 9**

**Table A3**

*Model overview*

| Dependent variable(s) | Model | Interactions^a^ |
| --- | --- | --- |
| Stereotypes (Studies 1 and 2), competence dimensions (Study 3), vote intention (Studies 1, 2, and 3) | Model 1 | - |
|  | Model 2 | Participant education * candidate education  Participant education * candidate political orientation |
|  | Model 3^b^ | Participant education * educational salience * candidate education  Participant education * educational salience * candidate political orientation |
|  | Model 4 | Participant education * educational identification * candidate education  Participant education * educational identification * candidate political orientation |
| Vote intention | Model 5 | - |
|  | Model 6 | Stereotype * participant education |

*Note.* All models include the interaction candidate education * candidate political orientation, and the control variables age, gender, and ethnic prejudice (and law and order, environmentalism in studies 1 and 2).

^a^ All interactions also include all lower order interactions (e.g. participant education * educational identification in model 4) and main effects.

^b^ Model 3 only presented in Supplemental Material (Appendix 11).

**Appendix 10**

**Table A4**

*Significant higher order interactions*

| Model^a^ | Dependent variable | Higher order interaction | Subgroup | b | se | p |
| --- | --- | --- | --- | --- | --- | --- |
| Pilot study | | | | | | |
| Model 2 | Vote intention | Candidate education*candidate political orientation*subjective education |  | -0.168 | 0.084 | 0.045 |
| Model 4 | Warmth | Candidate education*candidate political orientation*subjective education*educational identification |  | 0.550 | 0.222 | 0.013 |
| Study 2 | | | | | | |
| Model 4 | Theoretical competence | Candidate education*candidate competence*participant education*educational identification | Higher educated | -0.229 | 0.106 | 0.030 |

*Note.* In Study 3 participant education has three levels, *subgroup* refers to which contrast (reference category is ‘less educated’) was significant.

^a^ For model specification, see Appendix 7.

**Appendix 11: Results of the educational salience manipulation**

Study 1 showed significant three-way moderation with candidate education * participant education * educational salience for competence (b = 0.161; p = 0.038) and agency (b = 0.159; p = 0.016). These moderations indicate that when education is salient, the extent to which higher educated participants are more positive about the competence and agency of the higher educated candidates than less educated participants is stronger when education is salient than when not salient. That is, saliency increase the ingroup bias of higher educated, compared to less educated participants. These results were not replicated in Study 2; none of the three-way interactions were significant.

**Appendix 12: Additional measures**

Pilot study

1. Extent to which the participants share the same vision on society as the candidate
2. Five items on the respondent’s perception of the assessment and vote intention of the average Dutchman towards the four profiles
3. Item on whether the respondent had a specific political party in mind for the last seen respondent
4. Educational conflict scale
5. Political efficacy (internal and external)
6. Party vote intention
7. Perceived representativity of the Dutch parliament
8. Sense of entitlement scale
9. Parental education level
10. Expected educational level
11. Most important factor in evaluation of the candidates
12. Did profiles make respondent think of political party?
13. Was the political orientation relevant in assessing candidate?
14. Was the educational level relevant in assessing candidate?

Study 1

1. Employment status
2. Extent to which the participants share the same vision on society as the candidate
3. Item on whether the respondent had a specific political party in mind for the last seen respondent
4. Educational conflict scale
5. Political efficacy (internal and external)
6. Party vote intention
7. Perceived representativity of the Dutch parliament
8. Sense of entitlement scale
9. Parental education level
10. Most important factor in evaluation of the candidates
11. Did profiles make respondent think of political party?
12. Was the political orientation relevant in assessing candidate?
13. Was the educational level relevant in assessing candidate?
14. Attitudes towards the elite

Study 2

1. Extra stereotype traits: devoted and honest
2. Willingness to politically participate
3. Political efficacy (internal and external)
4. Educational conflict scale
5. Party vote intention
6. Perceived representativity of the Dutch parliament
7. Sense of entitlement scale
8. Parental education level
9. Most important factor in evaluation of the candidates
10. Did profiles make respondent think of political party?
11. Was the professional (political and societal) experience relevant in assessing candidate?
12. Was the educational level relevant in assessing candidate?

**Appendix 13: Results for the middle educated**

All coefficients reported here are differences between middle and less or higher educated, positive (negative) coefficients indicate that the middle educated have a higher (lower) coefficient than the less/higher educated.

In Study 1, we found no significant differences in the assessment of higher and less educated candidates between the middle and less educated. The middle educated differed significantly from the higher educated for competence (b = -0.201, p < 0.001), agency (b = -0.083, p = 0.002), shared identity (b = -0.233, p < 0.001), vote intention (b = -0.190; p < 0.001). While differences were only significant between the middle and higher educated, the middle educated generally had lower coefficients than both the higher and less educated (though since higher educated usually have higher coefficients than the less educated, differences were significant between higher and middle, but not middle and less educated). Moderating these relationships by educational identification showed a similar pattern. The moderation was weaker for middle educated than for higher educated for competence (b = -0.261, p < 0.001), agency (b = -0.149, p = 0.011), shared identity (b = -0.247, p < 0.001), vote intention (b = -0.196; p = 0.003). No significant differences between middle and less educated. For none of the dependent variables had educational identification a moderating effect on the assessment of candidates, similar to the less educated.

In Study 2, the middle educated differed only in the assessment of higher and less educated candidates on theoretical, rhetorical, and vote intention from either the less or the higher educated. For rhetorical competence, the middle educated differed significantly from the less educated (b = 0.116, p = 0.003), such that middle educated perceived a stronger difference in favor of higher educated candidates in rhetorical competence, similarly to higher educated (b = -0.006, p = 0.845). For theoretical competence, the middle educated held a middling position between less (b = 0.146, p < 0.001) and higher educated (b = -0.090, p = 0.005).The same pattern we see for vote intention (b_less educated_ = 0.106, p = 0.013; b_higher educated_ = -0.139, p < 0.001). The moderation of these relationship by identification showed no significant differences between the middle and the less or higher educated, except for vote intention, where the difference in strength of this moderation was weaker for middle educated than for higher educated (b = -0.148, p = 0.035). Overall, little consistent patterns were found for how the middle educated differs from the less or higher educated, they tend to have a middle position in strength of coefficients, but this is not always the case: more often the differences are insignificant.

**Appendix 14: Cell means, SD, n, and correlations between measurements**

**Pilot study**

**Cell means, SDs, Ns**

| Stereotype trait | Less educated | | | | | | Higher educated | | | | | |
| --- | --- | --- | --- | --- | --- | --- | --- | --- | --- | --- | --- | --- |
|  | Progressive | | | Conservative | | | Progressive | | | Conservative | | |
|  | M | SD | N | M | SD | N | M | SD | N | M | SD | N |
| Capable | 4.16 | 1.20 | 229 | 3.98 | 1.19 | 228 | 5.40 | 1.01 | 229 | 5.06 | 1.10 | 228 |
| Intelligent | 4.07 | 1.20 | 229 | 3.86 | 1.25 | 229 | 5.96 | 0.89 | 229 | 5.80 | 0.95 | 229 |
| Friendly | 5.34 | 0.91 | 229 | 4.70 | 1.24 | 229 | 5.36 | 0.90 | 229 | 4.68 | 1.22 | 228 |
| Sympathetic | 5.29 | 0.91 | 229 | 4.52 | 1.31 | 228 | 5.41 | 0.89 | 229 | 4.64 | 1.27 | 229 |
| Decisive | 4.42 | 1.26 | 229 | 4.66 | 1.28 | 228 | 5.02 | 1.11 | 229 | 5.20 | 1.15 | 229 |
| Confident | 4.71 | 1.08 | 228 | 4.90 | 1.09 | 229 | 5.10 | 1.02 | 229 | 5.30 | 1.01 | 228 |
| Honest | 4.98 | 0.99 | 229 | 4.95 | 1.10 | 229 | 5.19 | 0.97 | 229 | 5.03 | 0.98 | 229 |
| Trustworthy | 4.88 | 1.08 | 229 | 4.48 | 1.27 | 229 | 5.23 | 1.03 | 229 | 4.98 | 1.11 | 228 |
| Identifying | 3.80 | 1.44 | 229 | 3.30 | 1.38 | 228 | 4.68 | 1.38 | 229 | 4.17 | 1.41 | 229 |
| Recognizing | 3.85 | 1.48 | 228 | 3.25 | 1.39 | 228 | 4.51 | 1.35 | 229 | 4.16 | 1.44 | 229 |
| Vote intention | 4.55 | 2.36 | 229 | 3.48 | 2.12 | 229 | 6.24 | 2.21 | 229 | 5.16 | 2.49 | 229 |

**Correlations between measurements**

|  | Capable | Intelligent | Friendly | Sympathetic | Decisive | Confident | Honest | Trustworthy | Identifying | Recognizing | Vote intention |
| --- | --- | --- | --- | --- | --- | --- | --- | --- | --- | --- | --- |
| Capable | 1 |  |  |  |  |  |  |  |  |  |  |
| Intelligent | 0.68 | 1 |  |  |  |  |  |  |  |  |  |
| Friendly | 0.32 | 0.26 | 1 |  |  |  |  |  |  |  |  |
| Sympathetic | 0.35 | 0.31 | 0.78 | 1 |  |  |  |  |  |  |  |
| Decisive | 0.48 | 0.43 | 0.08 | 0.09 | 1 |  |  |  |  |  |  |
| Confident | 0.39 | 0.34 | 0.08 | 0.15 | 0.59 | 1 |  |  |  |  |  |
| Honest | 0.38 | 0.30 | 0.56 | 0.51 | 0.33 | 0.302 | 1 |  |  |  |  |
| Trustworthy | 0.53 | 0.48 | 0.61 | 0.57 | 0.36 | 0.327 | 0.64 | 1 |  |  |  |
| Identifying | 0.48 | 0.48 | 0.37 | 0.43 | 0.30 | 0.224 | 0.30 | 0.442 | 1 |  |  |
| Recognizing | 0.44 | 0.45 | 0.34 | 0.41 | 0.27 | 0.232 | 0.23 | 0.3938 | 0.8487 | 1 |  |
| Vote intention | 0.47 | 0.48 | 0.36 | 0.43 | 0.22 | 0.197 | 0.31 | 0.4104 | 0.5405 | 0.5257 | 1 |

**Study 1**

**Cell means, SDs, Ns**

| Stereotype trait | Less educated | | | | | | Higher educated | | | | | |
| --- | --- | --- | --- | --- | --- | --- | --- | --- | --- | --- | --- | --- |
|  | Progressive | | | Conservative | | | Progressive | | | Conservative | | |
|  | M | SD | N | M | SD | N | M | SD | N | M | SD | N |
| Capable | 4.59 | 1.14 | 695 | 4.65 | 1.15 | 695 | 5.04 | 1.07 | 694 | 5.05 | 1.11 | 694 |
| Intelligent | 4.66 | 1.10 | 695 | 4.61 | 1.10 | 694 | 5.43 | 1.04 | 693 | 5.40 | 1.04 | 694 |
| Friendly | 5.01 | 1.04 | 695 | 4.89 | 1.10 | 695 | 5.00 | 1.00 | 695 | 4.88 | 1.07 | 694 |
| Sympathetic | 4.98 | 1.08 | 695 | 4.93 | 1.11 | 695 | 5.03 | 1.06 | 694 | 4.93 | 1.13 | 694 |
| Decisive | 4.77 | 1.16 | 695 | 4.89 | 1.13 | 695 | 4.95 | 1.11 | 692 | 5.13 | 1.11 | 694 |
| Confident | 4.87 | 1.05 | 695 | 4.97 | 1.03 | 695 | 5.05 | 1.08 | 694 | 5.20 | 1.07 | 694 |
| Honest | 4.86 | 1.08 | 695 | 4.95 | 1.07 | 695 | 4.89 | 1.09 | 692 | 5.00 | 1.08 | 694 |
| Trustworthy | 4.79 | 1.08 | 695 | 4.82 | 1.09 | 695 | 4.88 | 1.10 | 695 | 4.96 | 1.08 | 694 |
| Identifying | 3.94 | 1.51 | 695 | 4.19 | 1.47 | 695 | 4.03 | 1.48 | 695 | 4.37 | 1.52 | 695 |
| Recognizing | 3.90 | 1.49 | 695 | 4.16 | 1.46 | 695 | 4.00 | 1.49 | 695 | 4.31 | 1.56 | 695 |
| Vote intention | 3.77 | 1.61 | 693 | 4.00 | 1.75 | 692 | 4.04 | 1.62 | 695 | 4.39 | 1.69 | 694 |

**Correlations between measurements**

|  | Capable | Intelligent | Friendly | Sympathetic | Decisive | Confident | Honest | Trustworthy | Identifying | Recognizing | Vote intention |
| --- | --- | --- | --- | --- | --- | --- | --- | --- | --- | --- | --- |
| Capable | 1 |  |  |  |  |  |  |  |  |  |  |
| Intelligent | 0.77 | 1 |  |  |  |  |  |  |  |  |  |
| Friendly | 0.67 | 0.60 | 1 |  |  |  |  |  |  |  |  |
| Sympathetic | 0.69 | 0.62 | 0.81 | 1 |  |  |  |  |  |  |  |
| Decisive | 0.74 | 0.67 | 0.61 | 0.66 | 1 |  |  |  |  |  |  |
| Confident | 0.71 | 0.67 | 0.64 | 0.66 | 0.76 | 1 |  |  |  |  |  |
| Honest | 0.69 | 0.62 | 0.73 | 0.74 | 0.69 | 0.70 | 1 |  |  |  |  |
| Trustworthy | 0.75 | 0.67 | 0.75 | 0.75 | 0.71 | 0.69 | 0.80 | 1 |  |  |  |
| Identifying | 0.53 | 0.48 | 0.47 | 0.54 | 0.50 | 0.44 | 0.50 | 0.53 | 1 |  |  |
| Recognizing | 0.54 | 0.48 | 0.47 | 0.53 | 0.52 | 0.45 | 0.50 | 0.53 | 0.89 | 1 |  |
| Vote intention | 0.42 | 0.41 | 0.37 | 0.41 | 0.39 | 0.34 | 0.40 | 0.43 | 0.53 | 0.53 | 1 |

**Study 2**

**Cell means, SDs, Ns**

| Stereotype trait | Less educated | | | | | | Higher educated | | | | | |
| --- | --- | --- | --- | --- | --- | --- | --- | --- | --- | --- | --- | --- |
|  | Non-competent | | | Competent | | | Non-competent | | | Competent | | |
|  | M | SD | N | M | SD | N | M | SD | N | M | SD | N |
| Decisive | 4.01 | 1.17 | 815 | 4.85 | 1.15 | 813 | 4.58 | 1.07 | 813 | 5.06 | 1.06 | 814 |
| Hard working | 4.09 | 1.26 | 815 | 5.02 | 1.20 | 814 | 4.69 | 1.15 | 810 | 5.21 | 1.09 | 814 |
| Intelligent | 3.86 | 1.09 | 813 | 4.57 | 1.16 | 813 | 5.30 | 1.09 | 811 | 5.43 | 1.07 | 814 |
| Smart | 3.88 | 1.12 | 812 | 4.64 | 1.15 | 814 | 5.11 | 1.10 | 814 | 5.32 | 1.06 | 815 |
| Eloquent | 3.92 | 1.08 | 813 | 4.55 | 1.10 | 815 | 4.81 | 1.09 | 813 | 5.06 | 1.07 | 816 |
| Linguistically proficient | 3.90 | 1.02 | 814 | 4.42 | 1.10 | 814 | 4.96 | 1.12 | 813 | 5.11 | 1.07 | 815 |
| Empathic | 4.28 | 1.08 | 813 | 4.45 | 1.06 | 813 | 4.46 | 1.02 | 812 | 4.61 | 1.04 | 814 |
| Social | 4.70 | 1.19 | 813 | 4.99 | 1.12 | 813 | 4.81 | 1.08 | 814 | 4.99 | 1.09 | 816 |
| Tactical | 3.89 | 1.06 | 813 | 4.51 | 1.08 | 813 | 4.45 | 1.01 | 813 | 4.80 | 1.02 | 815 |
| Competitive | 3.93 | 1.17 | 815 | 4.57 | 1.15 | 812 | 4.49 | 1.18 | 811 | 4.89 | 1.09 | 816 |
| Vote intention | 3.08 | 1.54 | 812 | 3.97 | 1.69 | 814 | 3.85 | 1.56 | 815 | 4.61 | 1.71 | 813 |

**Correlations between measurements**

|  | Decisive | Hard working | Intelligent | Smart | Eloquent | Linguistically proficient | Empathic | Social | Tactical | Competitive | Vote intention |
| --- | --- | --- | --- | --- | --- | --- | --- | --- | --- | --- | --- |
| Decisive | 1 |  |  |  |  |  |  |  |  |  |  |
| Hard working | 0.76 | 1 |  |  |  |  |  |  |  |  |  |
| Intelligent | 0.66 | 0.66 | 1 |  |  |  |  |  |  |  |  |
| Smart | 0.68 | 0.69 | 0.85 | 1 |  |  |  |  |  |  |  |
| Eloquent | 0.66 | 0.63 | 0.70 | 0.72 | 1 |  |  |  |  |  |  |
| Linguistically proficient | 0.62 | 0.60 | 0.74 | 0.72 | 0.77 | 1 |  |  |  |  |  |
| Empathic | 0.53 | 0.51 | 0.47 | 0.48 | 0.53 | 0.52 | 1 |  |  |  |  |
| Social | 0.55 | 0.57 | 0.47 | 0.49 | 0.52 | 0.49 | 0.60 | 1 |  |  |  |
| Tactical | 0.66 | 0.63 | 0.63 | 0.63 | 0.66 | 0.64 | 0.55 | 0.51 | 1 |  |  |
| Competitive | 0.68 | 0.66 | 0.61 | 0.62 | 0.60 | 0.59 | 0.47 | 0.48 | 0.64 | 1 |  |
| Vote intention | 0.41 | 0.42 | 0.43 | 0.43 | 0.39 | 0.39 | 0.29 | 0.31 | 0.39 | 0.37 | 1 |
